# Novel Approach to Inter‐Onset‐Interval Ratio Uncovers Music‐Like Rhythmic Patterns in Budgerigar (*Melopsittacus undulatus*) Warble Song

**DOI:** 10.1111/nyas.70164

**Published:** 2025-12-16

**Authors:** Jeroen van der Aa, Günther Koliander, W. Tecumseh Fitch, Marisa Hoeschele

**Affiliations:** ^1^ Department of Behavioral and Cognitive Biology University of Vienna Vienna Austria; ^2^ Acoustics Research Institute Austrian Academy of Sciences Vienna Austria

**Keywords:** budgerigar, isochrony, methodology, musicality, parrot, rhythm, vocalizations

## Abstract

Rhythm is an essential part of human music. Recently, there has been a surge of interest in the production of rhythmicity in nonhuman animal vocalizations. Novel methods have found widespread rhythmic behaviors—including those with music‐like properties—among nonhuman animals. Parrots appear to be uniquely flexible and self‐motivated in engaging with rhythmic structures. Previous work has found evidence supporting rhythmic capabilities in the budgerigar, a small parrot; however, little is known about rhythmicity in their natural behavioral repertoire thus far. As such, we investigated the rhythmic structure of their complex learned warble song, developing an adapted statistical approach that addresses assumptions/biases found in other methods. After validating this method using human speech and song data, we found nonrandom and structured rhythmicity in the budgerigar warble song that shows similarities in rhythmicity to human music. We also identified two warble element pairs that seem to be essential for producing these budgerigar rhythms. The grouped rhythmic distributions observed in budgerigars appear to arise from different individual strategies, with differing uses of these element pairs among male individuals. These results, combined with earlier work, suggest that rhythmicity is an important aspect of budgerigar communication.

## Graphical abstract

Recent work on non‐human animal vocalizations has offered new insights into rhythmicity. In an attempt to improve upon methods commonly used in animal rhythm research, we investigated the rhythmic structure of budgerigar song. Our proposed statistical approach showed rhythmicity in human music but not language, and uncovered non‐random structured rhythms in budgerigar, with differing individual strategies. This demonstrates rhythmic behavior in budgerigars’ natural vocal repertoire for the first time.

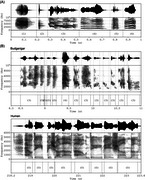

## 1 Introduction

Music is a ubiquitous human behavior, with rhythm as one of its core features supported by human biological predispositions [1, 2]. Rhythm manifests itself cross‐culturally through several human musical universals [3, 4]. Comparative research, utilizing the cognitively underlying traits of musicality, enables insights into the evolution of human rhythmicity, filling the gap caused by the lack of fossilization of behaviors [5].

Several rhythmic components of human musicality have been found in nonhuman animals in recent years. Entrainment (synchronization to an external stimulus) of motor behavior to external rhythms such as music is one of the human musical universals [4, 6]. Here, the evolutionarily distant parrots seem the most human‐like in their rhythmic motor capabilities, in particular. They are highly (self‐)motivated to align their movements (i.e., entrain) to different tempi of human music, a complex multidimensional acoustic construct from which a regular beat must be extracted, in a flexible manner in (short) bouts [7, 8, 9, 10]. The palm cockatoo (*Probosciger aterrimus*) is even known to use individualized and manufactured tools [11] in a flexible but consistent drumming display [12]. These results stand in stark contrast to our closest living relatives.

Nonhuman primates are quite limited in their entrainment capabilities, even to simple stimuli such as a metronome after extensive training [13, 14, 15, 16]. Chimpanzee (*Pan troglodytes*) drumming is often short and simple in structure [17], and although they select buttress roots to be used as tools in a nonrandom fashion [18], the chimpanzees do not modify these tools to suit the task. The only mammal described to reach flexible and highly accurate sustained entrainment to human music is a single pinniped (*Pinnipedia*). The California sea lion (*Zalophus californianus*) Ronan generalizes entrainment across tempi, while maintaining stable phase angles to the beat, after training with reinforcement [19, 20]. Moreover, harbor seal (*Phoca vitulina*) pups have been shown to produce antisynchronous entrainment in their natural vocal behavior [21]. Finally, while some crabs and different insect species are capable of entrainment, some with extremely high precision, this is limited to a small range of tempi (see Ref. [22] for an overview of species). This all suggests that, despite the evolutionary distance of parrots from humans, they might be more akin to human rhythmic musicality in their behavior than the more closely related mammalian species.

The budgerigar (*Melopsittacus undulatus*) is a parrot native to the dry inland of Australia and lives in large flocks. This common pet species is known for its complex learned vocalizations, called warble song, mostly produced by males [23, 24]. Previous work often focused on budgerigar auditory perception [25, 26, 27, 28, 29], including temporal features (e.g., order detection of species‐natural [26] or non‐natural [27] elements, and acoustic indicators of lexical stress [29]), but is lacking regarding the presence or nature of rhythmicity in their natural vocalizations. As for their rhythmic perception, budgerigars have been shown to be capable of entraining to auditory stimuli, albeit with limited precision after extensive training [30], and drift toward metronome distractors in a self‐paced tapping task [31]. Interestingly, a preference study indicated female budgerigars preferred stimuli with certain rhythmic features, such as isochrony (a pattern with equal intervals, e.g., a metronome), repeating motifs, and a steady beat, while males avoided these [32, 33]. Moreover, a recent study found correlational associations between different warble elements and motor action, including motions of a cyclical, repetitive, or rhythmic nature [34]. These results suggest that the budgerigar has rhythmic predispositions ingrained in their natural behavior, for which their complex learned warble song, whose temporal properties have so far eluded a detailed analysis due to its complexity [24, 35, 36], is a prime candidate.

A methodological approach that has recently garnered some popularity in the field of comparative research of rhythmicity is the investigation of the ratios of successive inter‐onset‐intervals (IOIs) in natural nonhuman animal vocalizations. The basic idea is that the IOI‐ratio can be used to describe the local rhythmic structure of any behavior, including structures common in human music cross‐culturally, for example, isochrony (metronome rhythm with 1:1 integer ratio, IOI‐ratio of 0.5), a 2:1 or 1:2 integer ratio (0.33 and 0.66) [37], or any other dyadic relationship on the IOI‐ratio spectrum of 0 to 1. First put forward by Roeske et al. [38], the method was used to demonstrate integer ratio rhythms in a songbird. Since then, studies using this method have led to novel insights into animal rhythmicity. This includes (1) context‐dependent isochrony in chimpanzee behavior [39] and differences in buttress drumming in chimpanzee subspecies [40], (2) embedded isochrony in the male vocalizations of an orangutan subspecies (*Pongo pygmaeus wurmbii*) [41], (3) isochronous [42, 43] or integer ratio rhythms [44] in different nongreat ape (*Hominoidea*) primate vocalizations, (4) context dependence of the rhythmicity of harbor seal vocalizations [45], and (5) isochronous vocalizations and its role in reproductive success in the rock hyrax (*Procavia capensis*) [46]. As such, IOI‐ratio distributions have the potential to describe previously undiscovered rhythms in animal behavior.

Despite all this recent work, statistical approaches using IOI‐ratios are not yet well established. A common strategy is to test whether IOI‐ratios in a specific region of interest (ROI; often around isochrony, i.e., 0.5) are significantly more frequent than in adjacent regions. This approach ignores the rest of the IOI‐ratio domain, and it is difficult to generalize to arbitrary ROIs. Specifically, moving away from the center of the IOI‐ratio domain, potential ROIs might get too close to each other, for example, testing for a local peak at relative IOIs 1:4 and 1:3 (corresponding to IOI‐ratios 0.20 and 0.25) necessitates ROIs much smaller than commonly used for testing isochrony (which are also not yet standardized). Moreover, simply describing a local peak in the IOI‐ratio distribution does not inform one about the mechanism underlying the rhythm, or whether it is more than expected by chance.

Potential mechanisms for some different rhythms could, for example, include motor production limitations (e.g., isochrony through a tempo ceiling) and innate stereotypies shaped by evolution as more constrained mechanisms. Vocal usage and vocal production learning (or learning of other nonvocal effectors) allow for changes in rhythmic production throughout life, but potentially also on a shorter timescale by dynamically changing rhythm production through (intentional) flexibility. One way to gain insights into the underlying mechanisms is by generating a null hypothesis model to which one can compare observed data. While some studies have not tested whether the detected effects could be explained by chance, other studies have compared their observed data to a random null hypothesis.

The common choice for a random null hypothesis consists of IOI‐ratios generated by a uniform distribution of IOIs over a finite interval. While this is generally argued to be a weak null hypothesis [47], we suggest that it is actually nonsensical. The IOIs in animal vocalizations are not expected to follow a uniform distribution. As species‐typical behavior differs from species to species, what constitutes random behavior will also vary from species to species. A random sampling of the intrinsic distribution of species‐natural behavior is an ideal comparison to observed data because it controls for exactly these species‐specific differences. Therefore, to avoid these issues, we propose a nonparametric approach that does not at all depend on any parametric modeling assumption or necessitates us to define a specific new IOI distribution. Instead, we randomize the observed data to generate novel IOIs for comparison.

The main goal of our current work is to explore whether rhythmic capabilities observed in experiments are rooted in the budgerigars’ natural behavioral repertoire, focusing on their warble song. Next to this, we aim to improve and expand upon previous methods applied to IOI‐ratio data and avoid some statistical pitfalls that earlier work is subject to, in particular regarding the uniform distribution null hypothesis. In order to assess our proposed methodology for the study of rhythmicity, we subject human data to the same methods. For this, we used the NUS‐HLT Speak‐Sing (NHSS) database, which consists of both sung and spoken recordings of 20 different pop songs with English lyrics [48]. Because the target lyrics are identical between the respective sung and spoken recordings, this database is controlled for word content and propositional meaning, and consists of (semi‐)natural human behavior. The NHSS database, therefore, allows us to compare the budgerigars to a balanced human dataset known to contain rhythmicity in the sung recordings, which here have been analyzed using the same methods as the budgerigar data. However, rhythm in nonhuman animals need not be limited to human rhythmic structures and organization. As such, we opt to take a broad perspective and definition of rhythm and rhythmicity, that is, any nonrandom (structured) temporal pattern of a (acoustic) signal.

## 2 Methods

### 2.1 Data Collection—Budgerigar

Recordings of 14 budgerigars (13M:1F) that were collected for earlier publications (all used in Ref. [49]) were analyzed. These recordings consist of four different budgerigar populations; eight birds (7M:1F) from two separate aviary populations (group A and B) at the Department of Cognitive Biology at the University of Vienna (now relocated to the Acoustics Research Institute of the Austrian Academy of Sciences), two pet birds housed in Arkansas, USA (group C), (all 10 birds recorded while freely moving in their familiar environment; see Ref. [49] for recording details), and four individuals recorded in isolation at the Laboratory of Comparative Psychoacoustics at the University of Maryland (see Refs. [26] and [50] for recording details, group D). Video recordings were used for individual identification when the birds were freely moving in a group setting, and warble song was extracted from the audio recordings based on a single individual vocalizing uninterrupted for at least 2.5 s (as in Ref. [49]). From the audio recordings, a total of 2 h, 7 min, and 5 s of warble song was extracted.

### 2.2 Data Collection—Human

Recordings of human vocalizations were obtained from the NHSS database, a collection of 200 audio recordings from 10 subjects (5M:5F) of sung and spoken English pop songs (10 songs per subject). We used the full audio recordings available in the database (not the recordings split per utterance) for our analyses. As such, we had a total duration of 6 h, 39 min, and 40 s available for the sung recordings, and 2 h, 41 min, and 24 s for the spoken recordings. See Ref. [48] for details on the recording process, descriptives, and some quantitative metrics of the NHSS database.

### 2.3 Data Collection—Ethics

All data were collected in accordance with local legislation, and data collection was approved by the relevant ethical boards for previous works. For the budgerigar data, ethical approval was obtained from either the ethical board of the behavioral research group in the faculty of Life Sciences at the University of Vienna (10 budgerigars; Approval number 2015–005), or the Animal Care and Use Committee of the University of Maryland (four budgerigars). Data collection and subsequent release of the human data was approved by the National University of Singapore (NUS).

### 2.4 Data Preparation—Preprocessing and Element Extraction

To improve signal‐to‐noise ratio, all audio recordings were preprocessed in a custom‐written Python script (v3.11.4, https://www.python.org), run in jupyterlab (v4.3.4, https://jupyter.org) using Anaconda (conda v23.7.4, https://www.anaconda.org). A bandpass filter was first applied (Butterworth bandpass; order = 5, SciPy v1.11.1, https://scipy.org) with a low–high threshold range of 400 Hz—15 kHz for the budgerigar recordings, and 75 Hz—5 kHz for the human recordings. To further reduce the noise from frequencies overlapping with the signal, we applied a Stationary Noise Reduction algorithm (noisereduce package v4.0.0 [51]; *reduce_noise* function, https://pypi.org/project/noisereduce/), where we set the noise threshold at ⅓ and 1 standard deviations above the mean for the budgerigar and human recordings, respectively.

**FIGURE 1:**
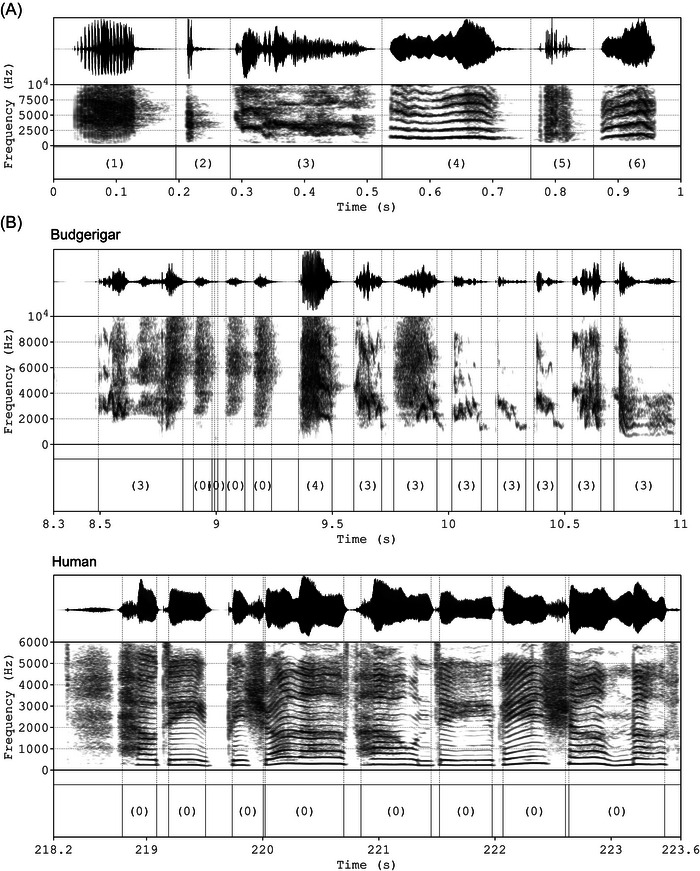
(A) Examples of the different element types extracted from budgerigar recordings. (B) Example segments of budgerigar and human recordings after preprocessing and element extraction. Since elements were not classified for the human recordings, they are all labeled as (0). Upper timeseries: (normalized) amplitude. Middle timeseries: spectrogram. Lower timeseries: element classifications with boundaries (start and end) as dotted lines over the other timeseries. Number coding for the element classifications: (1): *alarm*, (2): *click*, (3): *complex phrase*, (4): *long harmonic*, (5): *noisy*, (6): *short harmonic*, (0): *unknown*.

Using *parselmouth* (v0.4.5, https://pypi.org/project/praat‐parselmouth), a custom‐written Praat script was run on each preprocessed recording in order to extract the individual elements that were used for further analyses for both species. This script is an altered version of the one described in [49], using the intensity envelope by calculating the root‐mean‐square (RMS) of the sound pressure, where a reduction of the RMS below 1/6th of a recording's sound pressure level for a set duration was used as the criterion for element segmentation. For this study, we changed the RMS window length and time step to 5 and 1 ms, respectively, and the duration threshold to 5 ms in order to improve the temporal precision of the element boundary detection. The budgerigar elements were subsequently automatically identified according to the classification in Ref. [50] using the same script. Compound and contact call‐like elements were combined into a single “complex phrase” category, as in Ref. [49]. As such, we classified the following warble elements: *alarm*, *click*, *complex phrase*, *long harmonic*, *noisy*, and *short harmonic* (see Figure 1A for examples). These classifications were ignored for the human recordings. Afterwards, we cleaned our element and classification data by discarding errors. In particular, budgerigar *noisy* elements of a duration longer than 70 ms were reclassified as *unknown*, and elements deemed too short were discarded altogether (less than 10 ms for budgerigar elements, less than 20 ms for human elements). This error correction and all remaining downstream analyses were conducted in R (https://www.r‐project.org; v4.3.0), using RStudio (v2023.06.0+421). See Figure 1 for an example of both a budgerigar and a human recording with element annotations.

Before further analyzing the elements, they were grouped into bouts. Earlier research on human vocal turn‐taking has shown that language‐specific average delay ranges from 7.29 to 468.88 ms [52]. Based on this, we implemented a threshold of 500 ms of silence between elements as a cut‐off value for individual bouts in our human data. Visual inspection using this cut‐off showed bout‐boundaries at the end of sentences and within sentences (comma or hesitation). Because the distribution of silences between elements of the human data was similar to that of the budgerigars multiplied by a factor of 2, we applied a silence threshold of 250 ms for the budgerigar bouts. Using these methods, we isolated 4571 bouts (average of 8.90 elements per bout, range 1–657) across all budgerigars, and 3302 (average of 4.99 elements per bout, range 1–69) and 3300 bouts (average of 6.19 elements per bout, range 1–59) for the human sung and spoken recordings, respectively. These contained a total of 40,688 elements with 32,620 IOI‐ratios from the budgerigar recordings, and 16,467 and 20,424 elements with 10,246 and 13,955 IOI‐ratios from the human sung and spoken recordings, respectively.

### 2.5 Data Preparation—IOI‐Ratio

We define the IOI‐ratio as:

$$IOI-ratio_{k}=\frac{IOI_{k}}{IOI_{k}+IOI_{k-1}},$$

which is a slight deviation from the previous literature (in [38], it has been defined as the ratio between the current and the sum of current and next IOI, whereas we use the sum of the current and the previous IOI). This revised IOI‐ratio describes rhythmic patterns based on preceding events rather than future events and thus more closely aligns to the cognitive mechanisms underlying perception and production [53]. In particular, phenomena such as error correction in production and surprisal due to syncopation in perception are contingent on the instantiation of the rhythm into the physical (acoustic) domain. As a descriptive metric, this change has limited consequences for the distribution of the IOI‐ratio since it is simply mirrored (i.e., *old* IOI‐ratio = 1 − *revised* IOI‐ratio). However, depending on one's analyses using IOI‐ratio, this change might have consequences for the conclusions, and we argue that this revised IOI‐ratio (hereafter IOI‐ratio) offers insights that hold higher relevance from the perspective of both perception and production.

### 2.6 Data Analysis—Permutations and Statistics

Going forward, our null hypothesis is that the order of specific IOIs is random, and thus any IOI‐ratio distribution calculated from these would describe random behavior as well. For the budgerigar warble song specifically, this null hypothesis suggests that IOIs are exchangeable within element classes and individuals, that is, exchanging IOIs within these does not affect the IOI‐ratio distribution (note that each IOI‐ratio is calculated from neighboring IOIs and, therefore, exchanging IOIs would affect IOI‐ratios only if the IOIs are in a non‐random order). In contrast, if we reject the null hypothesis, this implies a structured ordering of IOI‐ratios to produce rhythmicity (or deliberately avoid certain rhythmic structures).

Our nonparametric statistical analysis was based on a permutation test [54], that is, we compared the IOI‐ratio distribution of the original IOI sequence with a large number (in our case 10,000) of IOI‐ratio distributions of random permutations of the original IOI sequence. Here, a random permutation corresponds to a reordering of the IOI sequence and thus leads to different IOI‐ratios. If the order of IOIs was determined by chance, then we would expect that the permutations would not change our overall distribution of IOI‐ratios compared to the original sequence. As per our null hypothesis, we only considered permutations within individual budgerigars and element classes. This approach means that the IOI‐ratios of the permuted IOI sequences are ratios between IOIs associated with the same element classes and individuals as in the original IOI sequence. Similarly, the IOIs in the human data were permuted within subjects. Song and speech data were analyzed independently so that we could compare between these two domains.

To build a test statistic that we can test against our null hypothesis, we first derived the IOI‐ratio distribution for the original IOI sequence and for each permutation using a Gaussian kernel density estimator (binwidth = 0.01, number of estimates = 512, stats v4.3.0). This provides us with a standardized number of occurrences (probability density estimate) of each IOI‐ratio at 512 IOI‐ratio values. To avoid multiple comparison issues, we constructed a single statistic from each IOI‐ratio distribution. More concretely, we first derived a z‐score‐like normalization (deviation) at each of 512 IOI‐ratio values by subtracting the mean and subsequently dividing by the standard deviation across all permutations (including for the original sequence). We then considered the maximum absolute deviation (i.e., the largest deviation while ignoring whether the deviation was positive or negative) of this “normalized deviation from the mean” across all IOI‐ratios for each permutation. The resulting “maximal normalized deviation from the mean” value is our test statistic. This test statistic should be low for an observed distribution that is close to the average of all permuted distributions, whereas it should be high for a distribution that is unique. We decided to use a normalized test statistic in order to avoid introducing biases toward some ratios over others (e.g., isochrony, which is very likely to occur by chance if many IOIs are close to average length), as they inherently have greater variation.

In order to test whether our empirically observed IOI‐ratio distribution from the original sequences of budgerigar, speech, and song data differed statistically significantly from the permuted data, we assessed whether the observed maximal normalized deviation from the mean was larger than 99% of the maxima observed for the 10,000 random permutations (i.e., we set an alpha of 0.01) in order to determine whether we can reject our null hypothesis. In other words, for each permuted distribution and for the observed distribution, we calculated the maximal normalized deviation from the mean, and we compared whether the observed distribution maximal normalized deviation value (a single value) was greater than that for 99% of the individual permuted distributions. The reason for this is that, by chance, we would expect some of these distributions also to show patterns that deviate from the average, but the majority of them, being randomly generated, should show patterns that are similar to the average across all distributions. If our observed data deviates, this would suggest that the order of IOIs is likely to be important.

Following rejection of the null hypothesis, we assessed specific effects across the IOI‐ratio distribution by using the confidence interval as a threshold. This confidence interval was calculated as the permutation data's 99% quantile range of the deviation across the IOI‐ratio distribution. Local peaks that exceed this confidence interval were identified using the *findpeaks* function (pracma package v2.4.4, https://cran.r‐project.org/web/packages/pracma/index.html).

## 3 Results

### 3.1 Human Recordings

**FIGURE 2:**
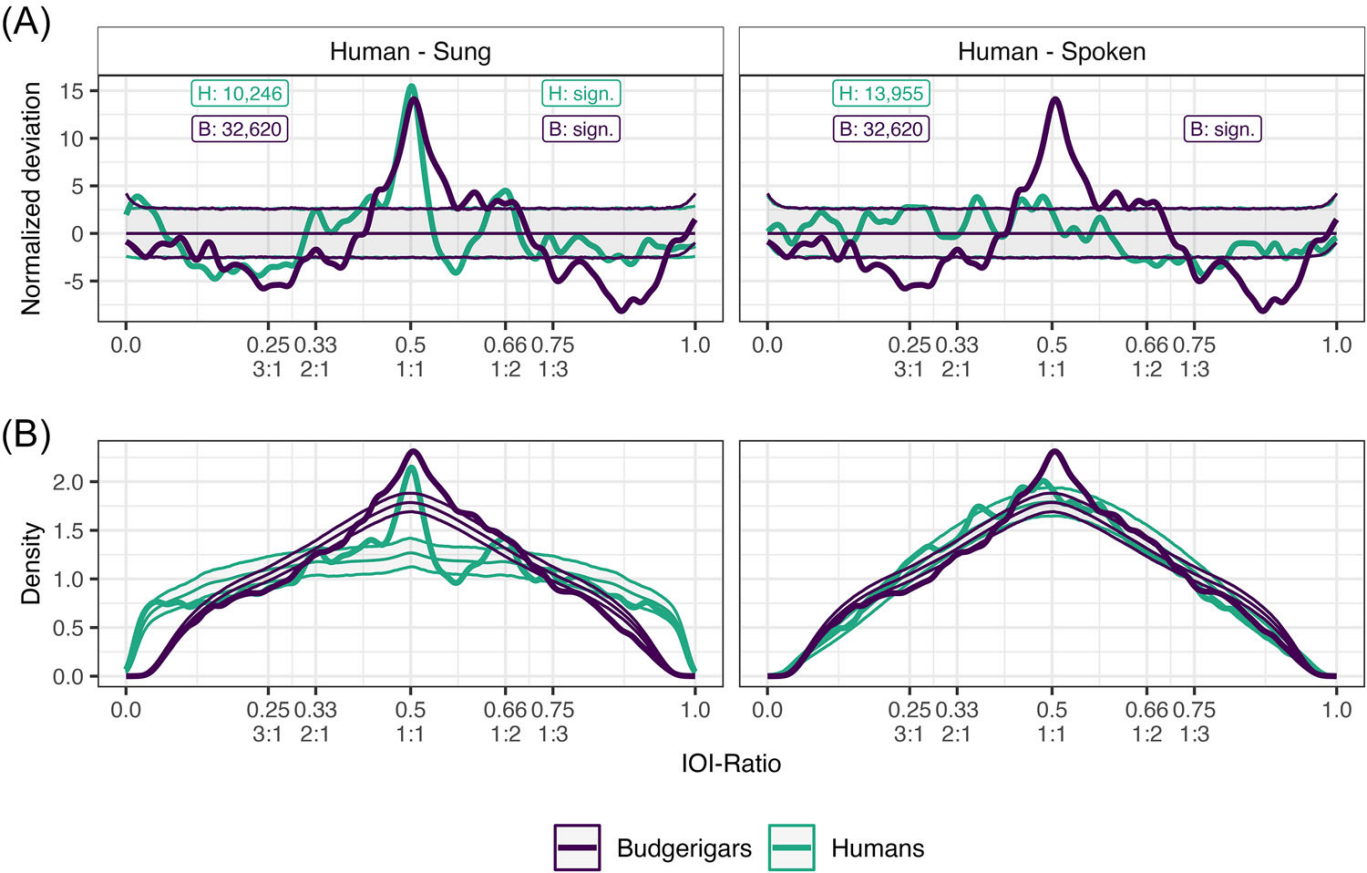
Grouped normalized deviation (A) and full density distributions (B) for both human and budgerigar data. Human sung recordings are displayed in the left column, and spoken recordings are displayed in the right column. Budgerigar data is identical for both columns. The thick line constitutes the observed data. The three thin lines constitute the permutation distributions: permutation average (middle line) ± confidence intervals (outer lines). Budgerigar data is plotted in purple, and human data is plotted in teal. Critical to see here is that both the budgerigar data and the human sung data but not the human spoken data (thick lines) greatly exceed the permuted averages (thin lines). Text boxes display underlying inter‐onset‐interval (IOI)‐ratio sample size on the left, and significance status on the right for both human (H) and budgerigar (B) data. Integer ratios are denoted below their respective IOI‐ratio. Note that the deviation plotted in (A) is the result of subtracting the permutation results from the observed distribution visible in (B) following further normalization.

For our human data, we found evidence for rhythmicity in the sung recordings, as opposed to the spoken recordings, which showed no significant rhythmicity (Table 1). For the sung recordings, we specifically find more than expected IOI‐ratios (positive deviation above the confidence interval threshold) at 0.50 (1:1), 0.667 (1:2), 0.431, and 0.020. We also find fewer than expected IOI‐ratios (negative deviation below the confidence interval threshold) from 0.106 to 0.301, and at 0.579, 0.718, 0.847, and 0.886 (Figure 2A). These results suggest structured and nonrandom rhythms in the NHSS sung recordings, specifically for the integer ratios of 1:1 and 1:2 (2:1 just barely below the confidence interval). Spoken recordings did not contain rhythmicity, as expected. Based on these results, we conclude our approach to be valid, and as such, applied it to the budgerigar data.

**TABLE 1:** Results of the statistical analyses for both the human and budgerigar data.

***Human***			
**Recording**		**Test statistic**	**Max. deviation**
Song		3.978287	15.469428 *
Speech		4.411807	4.356293
***Budgerigar***			
**ID (M/F)**	**Group**	**Test statistic**	**Max. deviation**
Grouped		4.367826	14.11123 *
AFI (M)	A	6.482440	2.535056
BEN (M)	A	8.128226	2.979747
BOB (M)	C	5.769965	4.292872
D18 (M)	D	8.081323	2.535557
ELV (M)	A	5.714861	6.146125 *
GAN (M)	A	8.145983	3.990322
HED (F)	A	6.601890	4.834083
MAY (M)	C	5.604473	6.127583 *
MER (M)	A	6.592522	4.332774
PUC (M)	A	5.141417	7.887247 *
PUF (M)	D	6.496953	4.208196
RIC (M)	D	7.724973	5.703882
TOP (M)	B	8.380697	4.119802
UM1 (M)	D	6.895641	4.108112

*Note*: Budgerigar sex is indicated in parentheses. The test statistic is equal to 99% of the maxima observed for the permutations. Maximum deviations of the observed data that are larger than the test statistic are demarcated with an asterisk (*).

Abbreviations: F, female; M, male.

### 3.2 Budgerigar Recordings

For our budgerigar data, we found evidence for rhythmicity (Table 1). Specifically, we found more than expected IOI‐ratios from 0.432 to 0.630 (notable peaks at 0.505, 0.446, and 0.612), and from 0.638 to 0.689 (notable peaks at 0.651 and 0.677). We also find fewer than expected IOI‐ratios at 0.033, 0.123, from 0.160 to 0.319 (notable peaks at 0.172, 0.241, and 0.282), at 0.376, and from 0.742 to 0.947 (notable peaks at 0.759, 0871, and 0.904, Figure 2A). Based on these results, we conclude that budgerigar warble song contains structured rhythmicity, specifically around the integer ratios of 1:1 (0.433−0.630 range, with a peak at 0.505) and 1:2 (0.638−0.689 range, with peaks at 0.651 and 0.677), similar to the results found in the human sung recordings. The rhythmicity described in the budgerigars is less precise and more variable than that described in the human sung recordings, and does not show any tendency for an effect at 2:1. Moreover, budgerigars show fewer IOI‐ratios for larger integer ratios (i.e., 1:3 and 3:1 and larger) than expected.

**FIGURE 3:**
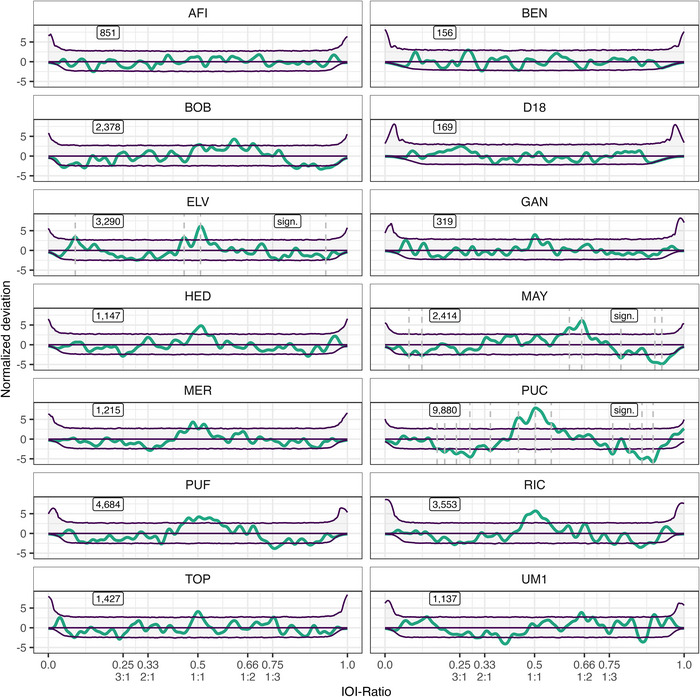
Normalized deviation distributions per budgerigar individual. The thick (teal) line constitutes the observed data. The three (purple) thin lines constitute the permutation distributions: permutation average (middle line) ± confidence intervals (outer lines). Dashed lines demarcate local peaks that exceed the confidence interval. Text boxes display underlying inter‐onset‐interval (IOI)‐ratio sample size on the left, and significance status on the right per individual. Integer ratios are denoted below their respective IOI‐ratio.

When looking at each individual budgerigar, we find rhythmicity in 3 out of 14 birds (all male, as we only had recordings of one female), specifically in Elvis (ELV, group A), Maya (MAY, group C), and Puck (PUC, group A) (Table 1). Note that all three of these individuals had large sample sizes (>3400 elements), and PUC had by far the largest sample in our set (12,091 elements). For ELV, we find more than expected IOI‐ratios at 0.509, 0.454, and 0.090 and fewer than expected at 0.928. For MAY, we find more than expected IOI‐ratios from 0.587 to 0.679 (notable peaks at 0.658 and 0.616), and fewer than expected at 0.080, 0.123, and 0.788, and from 0.877 to 0.965 (notable peaks at 0.902 and 0.926). Lastly, for PUC, we find more than expected IOI‐ratios from 0.429 to 0.569 (notable peaks at 0.503, 0.446, and 0.556), and fewer than expected from 0.170 to 0.303 (notable peaks at 0.200, 0.284, and 0.352), at 0.352 and 0.761, and from 0.808 to 0.916 (notable peaks at 0.859 and 0.896). See Figure 3 for all budgerigar individuals’ deviations.

The grouped budgerigar results described above seem to be caused by two different rhythmic strategies. Some individuals limit the nonrandom elements of the vocalization to isochrony (1:1), with some additional acceleration (e.g., ELV and PUC with acceleration IOI‐ratios at 0.454 and 0.446, respectively). One other individual (MAY) seemed to limit their nonrandom vocalization elements generally to local deceleration (IOI‐ratio greater than 0.5, i.e., a short‐long IOI pair), peaking just before the 1:2 integer ratio, while avoiding larger IOI‐ratios. A peak around isochrony was barely below the confidence interval for MAY, though.

**FIGURE 4:**
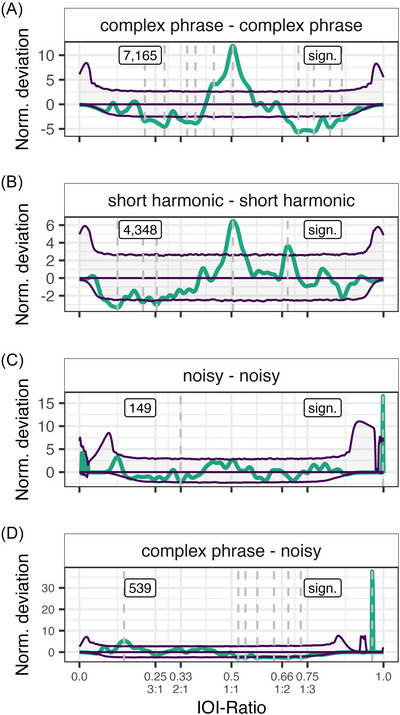
Normalized deviation distributions for the four significant element pairs. The thick (teal) line constitutes the observed data. The three (purple) thin lines constitute the permutation distributions: permutation average (middle line) ± confidence intervals (outer lines). Dashed lines demarcate local peaks that exceed the confidence interval. Text boxes display underlying inter‐onset‐interval (IOI)‐ratio sample size on the left, and significance status on the right per individual. Integer ratios are denoted below their respective IOI‐ratio.

When looking at all possible warble element pairs (excluding unknown) across individuals, we find rhythmicity in 4 out of 36 pairs, specifically complex phrase followed by a complex phrase (C‐C), complex phrase followed by a noisy (C‐N), noisy followed by a noisy (N‐N), and a short harmonic followed by a short harmonic (S‐S, Table S1). For the C‐C pair, we find more than expected IOI‐ratios at 0.505 and 0.442, and fewer than expected from 0.205 to 0.295 (notable peak at 0.280), and from 0.323 to 0.399, and from 0.677 to 0.885 (notable peaks at 0.702, 0.771, and 0.824, Figure 4A). For the S‐S pair, we find more than expected IOI‐ratios at 0.505 and 0.685, and fewer than expected at 0.254, 0.209, and 0.125 (Figure 4B). For the N‐N pair, we find more than expected IOI‐ratios at 0.998, and fewer than expected at 0.333 (Figure 4C). Lastly, for the C‐N pair, we find more than expected IOI‐ratios at 0.962 and 0.147, and lower than expected at 0.523 and 0.546, and from 0.577 to 0.750 (Figure 4D). See Figure S1 for all element pairs’ deviations and Figure S2 for the pairs’ IOI‐ratio densities and element descriptives.

Based on these element‐pair results, we conclude that mainly the C‐C and S‐S pairs are responsible for the effects observed in earlier analyses. These element‐pairs show nonrandom effects similar to the grouped budgerigar results, namely, an isochronous peak (C‐C and S‐S), an effect around 0.666 (S‐S), and fewer than expected IOI‐intervals above 1:2 (C‐C). In both noisy pairs, the sample size is on the lower end. Moreover, the main peaks observed are on the extreme end of the IOI‐ratio spectrum (0.998 and 0.962), where inflation of the normalized deviation may have occurred due to the very low standard deviation. This also means that the actual density of the IOI‐ratio peaks underlying the observed deviation is low as well (Figure S2). As such, we consider it unlikely that the effects we observed in these two noisy pairs are relevant.

**FIGURE 5:**
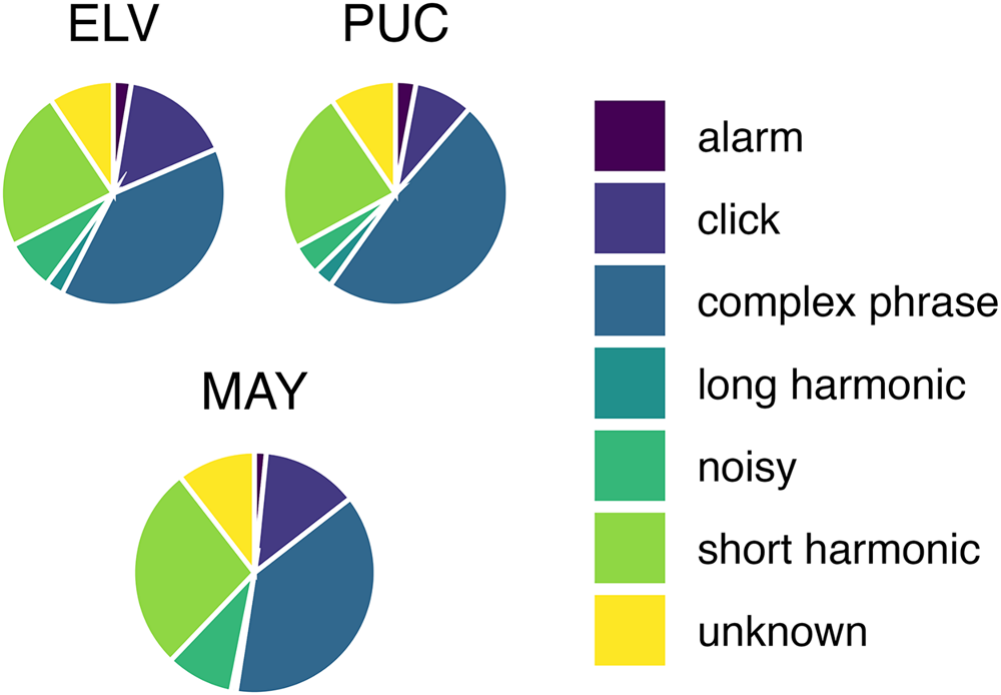
Distribution of different warble elements produced by the three budgerigar individuals who achieved significant rhythmicity.

While the distribution of elements within the above described individuals only seems to differ slightly (Figure 5 and Table 2), there was a noticeable difference between MAY and both ELV and PUC in the amount of the short harmonic elements present (27.38% vs. 23.23% and 23.41%, respectively, see Table S2 for all element per individual distributions), and between PUC and both ELV and MAY in the amount of complex phrase elements present (48.42% vs. 39.0% and 37.93%, respectively). It is possible these elements’ pair‐effects described above directly cause the distributions observed in these individuals, specifically C‐C for isochrony, and S‐S for isochrony and the region around 0.666. We, therefore, conducted a post‐hoc analysis where we redid all of our permutations and subsequent analyses for these three birds, where we omitted the S‐S and C‐C pairs specifically in order to assess the role these element‐pairs play in the production of these birds’ rhythms.

**TABLE 2:** Distribution of elements produced for the three significant budgerigar individuals, in percentages.

**ID**	**alarm**	**click**	**c. phrase**	**l. harm**.	**noisy**	**s. harm**.	**unkn**.
ELV	2.60%	15.92%	39.01%	2.58%	7.29%	23.23%	9.38%
MAY	1.59%	12.91%	37.93%	0.69%	9.02%	27.38%	10.49%
PUC	2.96%	8.44%	48.42%	2.83%	4.40%	23.41%	9.54%
AVG	2.30%	10.60%	40.40%	1.99%	6.17%	27.6%	11.0%

*Note*: The average distribution (AVG) is across all budgerigars recorded.

**FIGURE 6:**
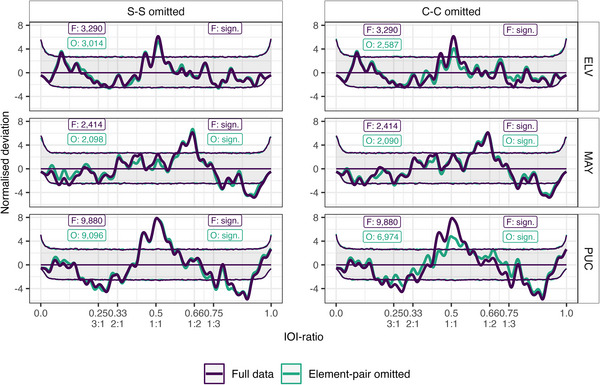
Normalized deviation distributions for per significant individual, for two post‐hoc element‐pair omissions, including the original (full) data without omission. The two different element‐pair omissions are displayed per column, and the three different individuals are displayed per row. The thick line constitutes the observed data. The three thin lines constitute the permutation distributions: permutation average (middle line) ± confidence intervals (outer lines). Original data is plotted in purple, and the analyses with omission are plotted in teal. Text boxes display underlying inter‐onset‐interval (IOI)‐ratio sample size on the left, and significance status on the right per individual per omission. Integer ratios are denoted below their respective IOI‐ratio.

When omitting the S‐S pair, MAY and PUC maintained significance, unlike ELV, with PUC in particular being barely affected by the omission (Figure 6 and Table S3). While ELV was no longer significant, the distribution similarly showed little changes, with most notably a small change lowering of the isochrony peak. Even though MAY maintained significance, the distribution was more strongly affected by the omission of the S‐S pair, but not where expected. Since S‐S was the pair with a peak at 0.685, we would have expected a larger effect around this IOI‐ratio. Instead, the “acceleration region,” originally from 0.587 to 0.679, was barely affected, even increasing marginally. Moreover, the region from ca. 0.400 to 0.550 seemed to be affected by this omission, even though the peaks here were originally below the confidence interval.

When omitting the C‐C pair, both MAY and PUC maintained significance once again (Figure 6 and Table S3). Despite PUC remaining significant, both ELV and PUC showed severely diminished amounts of IOI‐ratios around the isochronous region. On the other hand, MAY is barely affected by the C‐C omission. The results of these omissions thus show that the C‐C pair plays a large role in the rhythmic structure of the warble song for some individuals, with the S‐S pair also playing a role in some individuals. Moreover, different individuals use the elements in an individualized manner, which is not limited to element‐specific stereotypies to produce the individually differing rhythm of the song.

## 4 Discussion

In this work, we have put forward an adapted statistical approach to assess rhythmicity in natural vocalizations based on IOI‐ratio, and applied it to budgerigar vocalizations. Using this method, we found rhythmicity in the budgerigar song that shows similarities to the results from the human sung recordings. Moreover, we identified two element pairs (short harmonic followed by short harmonic and complex phrase followed by a complex phrase) that are often involved in this structured rhythmicity, and that different individuals use these element pairs in an individualized, nonstereotypical, manner to produce their individual rhythms.

Using a permutation analysis, we were able to compare natural song data to datasets that contained the same distribution of intervals between elements while controlling for the overall order and, therefore, the relative intervals of the songs. By normalizing the data across the IOI‐ratio spectrum (deviation), we were not limited to a single region of interest or parametric statistics. This allowed us to avoid biases about which kinds of rhythms may be relevant in other species, and the fact that the data provided its own control made it possible to exclude other potential reasons for finding rhythmicity. For example, when compared to a uniform distribution (i.e., a control where all intervals have the same likelihood of occurring), one might find isochronous (1:1) rhythmicity simply because in a natural distribution the average interval between elements occurs more often than in a uniform distribution (i.e., it is more likely in a natural distribution to get two intervals following one another that are close to average than in a uniform distribution where everything is equally likely).

In order to validate our approach, we applied these methods to recordings with known rhythmicity, human vocalizations. Our analyses returned the expected results, with nonrandom rhythmic structures in sung pop songs, and no evidence for nonrandom rhythmic structures in spoken pop song lyrics. Moreover, we found evidence for simple integer ratios in the sung recording, in particular 1:1 (IOI‐ratio 0.5) and 2:1 (IOI‐ratio 0.33), with 1:2 (IOI‐ratio 0.66) just barely remaining below the confidence interval. The fact that we find these effects, despite segmenting the human recordings using logic intended for budgerigar elements (onset detection based on the intensity envelope) and, therefore, most likely with limited accuracy and increasing noise (see Figure 1B for an example), further highlights the validity of this normalized deviation approach based on permutations.

Applying these methods to budgerigar warble song unveils rhythmicity that has eluded other researchers in the past [24, 36], possibly due to the fact that their temporal analyses were limited to element and IOI durations as opposed to relative metrics such as IOI‐ratio. In particular, we found rhythmicity in three male individuals where we had higher sample sizes, with at least two clearly differing focal areas. One area centered around isochrony with some minor acceleration (IOI‐ratio lower than 0.5), the second largely involved deceleration (IOI‐ratio greater than 0.5) up to the 1:2 integer ratio, while strongly avoiding larger IOI‐ratios. These ratios are also commonly found cross‐culturally in human music [4, 37], albeit often used interchangeably and recurring, and not as a single, individualized motif. While we also found two element pairs that played a role in the rhythmic structures, a short harmonic followed by a short harmonic and a complex phrase followed by a complex phrase, their influence on the rhythmic structure differed per individual. This implies that different individuals use different element pairs in their own unique fashion. If so, such individual specificity would suggest flexibility in how budgerigars implement rhythmicity. Given that budgerigar song is a learned vocalization [55], it makes sense that, as in different genres of human music, different songs from different individuals would be built from different rhythmic patterns.

The mechanisms underlying the effects described here remain unknown. Auditory feedback is known to be relevant for learning temporal characteristics of calls and warble elements [56], but the temporal properties of the warble song have also been suggested to be innate [57]. Here, it is unclear whether the rhythms found in the budgerigar vocalizations are intentional or occur due to stereotypy/motor constraints. However, given the flexibility and variability of budgerigar song as a learned vocalization, it is certainly possible that the rhythms observed here were produced for a functional purpose. Male budgerigars are known to modify their vocalizations based on the sex of the audience, including changes to the amount of contact call‐like elements (part of a complex phrase in the current study) and transition probabilities of short harmonic elements [58]. Moreover, male budgerigars incorporate aspects of their partner's warble song into their own, leading to the suggestion that budgerigar vocal imitation plays a role in sexual selection [59]. Combined with the fact that female budgerigars are known to prefer rhythmic features, while males avoid these [32, 33], we suggest that female preferences might be driving male warble behavior [60, 61, 62]. Our bias in mostly male recordings and the variability of their behavior aligns with this hypothesis. Moreover, it could be possible that the two differing rhythmic distributions are a result of the budgerigars dynamically changing the rhythm of their warble song because of an audience effect. Alternatively, since ELV and PUC are housed in the same aviary (group A), their similar warble song rhythms could be a consequence of a similar (acoustic) learning environment, as budgerigars are known to learn new vocalizations from conspecifics even in adulthood [63]. Either way, these suggestions merit further investigation.

While female budgerigars do produce warble song, they do so less and with a different organization compared to their male counterparts [24, 64]. As such, we only have one female budgerigar in our current sample, Hedwig, for whom we were unable to show rhythmicity, possibly due to their lower sample size. Many different functions for the warble song have been suggested, for example, social bonding, integration and cohesion of flock members, and individual and group recognition [24]. These might, therefore, also differ between sexes, and, similar to the audience effect described above, might impact the warble song structure. Taking the broader environment into account (including the audience) could, therefore, lead to novel insights regarding both the role and structure of the warble song within both individuals and sexes in future work. Because the contact call and contact‐like warble element have been suggested to be most relevant for individual and group recognition [65, 66], our results suggest that these processes might be facilitated by these warble elements’ rhythmicity.

While we were able to describe rhythmic structures in some individuals, this was limited to three out of the 14 sampled, all males. The fact that significant results were limited to those individuals with the highest sample size gives us reason to believe these results would generalize to the other individuals as well and could be due to power. However, this cannot be concluded based on the current work. A low sample size in our approach has two issues: first, we might not observe sufficient detail to get the true underlying probabilistic structure of each individual using permutation; second, that outliers play a stronger role also in the permuted versions, and thus the threshold (test statistic) becomes larger. Future work might be able to more clearly shed a light on the extent of music‐like rhythmic behavior throughout budgerigar populations and sexes.

Earlier work on the complex phrase elements has suggested that these units are potentially made from smaller segments that share similarities to consonants and vowels as observed in human language [49]. In the current study, the overall IOI‐ratio density distribution was more similar to the spoken human distribution, while the nonrandom deviation distribution was more similar to the sung distribution (Figure 2). Rhythmically, budgerigar warble song thus seems to contain aspects of both human song and speech. Warble song is music‐like in its ordering of temporal intervals between elements, and language‐like in the overall distribution of temporal intervals, as well as a similar fundamental unit structure. These nonrandom features do not appear to be as precisely aligned to specific integer ratios compared to music, and seem to arise from different underlying individual rhythms. One explanation for the differences observed would be the different use cases of human music as compared to budgerigar song, where one important aspect of music that is lacking in budgerigars is collaborative group performances (of singing), where synchronization is facilitated by rhythmical predictability through, for example, a reduction in vocabulary (strict integer ratios) and meter [67]. Finally, while we used the IOI‐ratio as a proxy measure for rhythmicity, we want to highlight that this measure is suited only for describing local (adjacent) effects and, as such, could benefit from alternative and complementary methods looking at larger time scales [68].

## 5 Conclusion

In this current work, we proposed a methodology that allowed us to directly compare Western music and budgerigar warble song without a strong preconception or bias. Not only do we find rhythmic structures in the budgerigar warble song in some male individuals, but these also show similarities with rhythmic patterns found in human music. These rhythms were individual‐, and possibly population (different aviaries that are maintained in acoustic isolation from one another) specific as for humans, something that has been described for other parrots [69]. By developing methods to determine whether other species nonrandomly and flexibly create rhythms in their natural vocalizations, we can understand what role rhythm plays in communication across species and thereby gain insights into the evolution of human music. Because we know that budgerigars can synchronize with rhythms and that female budgerigars prefer steady rhythms, this rhythmic information in warble song likely has communicative value in this species.

## Author Contributions

Conceptualization—J.vdA. and M.H. Methodology—J.vdA., G.K., and M.H. Data analysis—J.vdA. and G.K. Software—J.vdA. Validation—J.vdA, G.K., and M.H. Visualization—J.vdA. Writing—original draft—J.vdA. and G.K. Writing—review and editing—J.vdA., G.K., W.T.F., and M.H. Funding acquisition—W.T.F. and M.H.

## Funding

The authors declare that financial support was received for the research, authorship, and/or publication of this article. This article was supported by the Austrian Science Fund (FWF) DK Grant “Cognition & Communication 2” (#W1262‐B29) and the Vienna Science and Technology Fund (WWTF) project ANIML (LS23‐014). The funders had no role in the decision on the article content, the decision to publish, or the preparation of the manuscript.

## Conflicts of Interest

The authors declare that the research was conducted in the absence of any commercial or financial relationships that could be construed as a potential conflict of interest.

## Supporting information




**Supplementary Materials**: nyas70164‐sup‐0001‐SuppMat.pdf

## Data Availability

The datasets presented in this study can be found in online repositories. We have made the audio PRAAT textgrids, Python notebook, PRAAT scripts, and R markdown with related files available at the external repository PHAIDRA [https://phaidra.univie.ac.at/o:2172416]. This does not include the audio WAV files. Audio files of the budgerigar recordings can be provided upon request. The NHSS database and its information can be found at https://hltnus.github.io/NHSSDatabase/.

## References

[1] S. E. Trehub , “The Developmental Origins of Musicality,” Nature Neuroscience 6, no. 7 (2003): 669–673, 10.1038/nn1084. 12830157

[2] I. Winkler , G. P. Haden , O. Ladinig , I. Sziller , and H. Honing , “Newborn Infants Detect the Beat in Music,” Proceedings of the National Academy of Sciences of the United States of America 106, no. 7 (2009): 2468–2471, 10.1073/pnas.0809035106. 19171894 PMC2631079

[3] S. A. Mehr , M. Singh , D. Knox , et al., “Universality and Diversity in Human Song,” Science 366, no. 6468 (2019): eaax0868, 10.1126/science.aax0868. PMC7001657 31753969

[4] P. E. Savage , S. Brown , E. Sakai , and T. E. Currie , “Statistical Universals Reveal the Structures and Functions of Human Music,” Proceedings of the National Academy of Sciences of the United States of America 112, no. 29 (2015): 8987–8992, 10.1073/pnas.1414495112. 26124105 PMC4517223

[5] W. T. Fitch , “The Evolution of Music in Comparative Perspective,” Annals of the New York Academy of Sciences 1060 (2005): 29–49, 10.1196/annals.1360.004. 16597747

[6] S. Brown and J. Jordania , “Universals in the World's Musics,” Psychology of Music 41, no. 2 (2011): 229–248, 10.1177/0305735611425896.

[7] A. Schachner , T. F. Brady , I. M. Pepperberg , and M. D. Hauser , “Spontaneous Motor Entrainment to Music in Multiple Vocal Mimicking Species,” Current Biology 19, no. 10 (2009): 831–836, 10.1016/j.cub.2009.03.061. 19409786

[8] A. D. Patel , J. R. Iversen , M. R. Bregman , and I. Schulz , “Experimental Evidence for Synchronization to a Musical Beat in a Nonhuman Animal,” Current Biology 19, no. 10 (2009): 827–830, 10.1016/j.cub.2009.03.038. 19409790

[9] A. D. Patel , “Vocal Learning as a Preadaptation for the Evolution of Human Beat Perception and Synchronization,” Philosophical Transactions of the Royal Society B: Biological Sciences 376, no. 1835 (2021): 20200326, 10.1098/rstb.2020.0326. PMC8380969 34420384

[10] Y. Seki , “Cockatiels Sing Human Music in Synchrony With a Playback of the Melody,” PLoS ONE 16, no. 9 (2021): e0256613, 10.1371/journal.pone.0256613. 34478436 PMC8415583

[11] R. Heinsohn , C. N. Zdenek , D. Appleby , and J. A. Endler , “Individual Preferences for Sound Tool Design in a Parrot,” Proceedings of the Royal Society B Biological Sciences 290, no. 2006 (2023): 20231271, 10.1098/rspb.2023.1271. PMC10498050 37700644

[12] R. Heinsohn , C. N. Zdenek , R. B. Cunningham , J. A. Endler , and N. E. Langmore , “Tool‐Assisted Rhythmic Drumming in Palm Cockatoos Shares Key Elements of Human Instrumental Music,” Science Advances 3, no. 6 (2017): e1602399, 10.1126/sciadv.1602399. 28782005 PMC5489270

[13] E. Selezneva , S. Deike , S. Knyazeva , H. Scheich , A. Brechmann , and M. Brosch , “Rhythm Sensitivity in Macaque Monkeys,” Frontiers in Systems Neuroscience 7 (2013): 49, 10.3389/fnsys.2013.00049. 24046732 PMC3764333

[14] Y. Hattori , M. Tomonaga , and T. Matsuzawa , “Spontaneous Synchronized Tapping to an Auditory Rhythm in a Chimpanzee,” Scientific Reports 3 (2013): 1566, 10.1038/srep01566. 23535698 PMC3610097

[15] W. Zarco , H. Merchant , L. Prado , and J. C. Mendez , “Subsecond Timing in Primates: Comparison of Interval Production Between Human Subjects and Rhesus Monkeys,” Journal of Neurophysiology 102, no. 6 (2009): 3191–3202, 10.1152/jn.00066.2009. 19812296 PMC4073907

[16] H. Merchant and H. Honing , “Are Non‐Human Primates Capable of Rhythmic Entrainment? Evidence for the Gradual Audiomotor Evolution Hypothesis,” Frontiers in Neuroscience 7 (2014): 274, 10.3389/fnins.2013.00274. 24478618 PMC3894452

[17] V. Eleuteri , M. Henderson , A. Soldati , G. Badihi , K. Zuberbühler , and C. Hobaiter , “The Form and Function of Chimpanzee Buttress Drumming,” Animal Behaviour 192 (2022): 189–205, 10.1016/j.anbehav.2022.07.013.

[18] M. Fitzgerald , E. P. Willems , A. Gaspard Soumah , T. Matsuzawa , and K. Koops , “To Drum or Not to Drum: Selectivity in Tree Buttress Drumming by Chimpanzees (*Pan troglodytes* verus) in the Nimba Mountains, Guinea,” American Journal of Primatology 84, no. 7 (2022): e23382, 10.1002/ajp.23382. 35383993 PMC9540414

[19] P. Cook , A. Rouse , M. Wilson , and C. Reichmuth , “A California Sea Lion (*Zalophus californianus*) Can Keep the Beat: Motor Entrainment to Rhythmic Auditory Stimuli in a Non Vocal Mimic,” Journal of Comparative Psychology 127, no. 4 (2013): 412–427, 10.1037/a0032345. 23544769

[20] P. F. Cook , C. Hood , A. Rouse , and C. Reichmuth , “Sensorimotor Synchronization to Rhythm in an Experienced Sea Lion Rivals That of Humans,” Scientific Reports 15, no. 1 (2025): 12125, 10.1038/s41598-025-95279-1. 40312403 PMC12045976

[21] A. Ravignani , “Timing of Antisynchronous Calling: A Case Study in a Harbor Seal Pup (*Phoca vitulina*),” Journal of Comparative Psychology 133, no. 2 (2019): 272–277, 10.1037/com0000160. 30550302

[22] M. D. Greenfield and B. Merker , “Coordinated Rhythms in Animal Species, Including Humans: Entrainment From Bushcricket Chorusing to the Philharmonic Orchestra,” Neuroscience and Biobehavioral Reviews 153 (2023): 105382, 10.1016/j.neubiorev.2023.105382. 37673282

[23] B. F. Brockway , “Ethological Studies of the Budgerigar: Reproductive Behavior,” Behaviour 23, no. 3–4 (1964): 294–323, 10.1163/156853964X00193.

[24] S. M. Farabaugh , E. D. Brown , and R. J. Dooling , “Analysis of Warble Song of the Budgerigar (*Melopsittacus undulatus*),” Bioacoustics 4, no. 2 (1992): 111–130, 10.1080/09524622.1992.9753211.

[25] R. J. Dooling , S. D. Brown , T. J. Park , K. Okanoya , and S. D. Soli , “Perceptual Organization of Acoustic Stimuli by Budgerigars (*Melopsittacus undulatus*): I. Pure Tones,” Journal of Comparative Psychology 101, no. 2 (1987): 139–149, 10.1037/0735-7036.101.2.139. 3608422

[26] H. W. Tu and R. J. Dooling , “Perception of Warble Song in Budgerigars (*Melopsittacus undulatus*): Evidence for Special Processing,” Animal Cognition 15, no. 6 (2012): 1151–1159, 10.1007/s10071-012-0539-1. 22890832 PMC3474876

[27] M. J. Spierings and C. ten Cate , “Budgerigars and Zebra Finches Differ in How They Generalize in an Artificial Grammar Learning Experiment,” Proceedings of the National Academy of Sciences of the United States of America 113, no. 27 (2016): E3977–3984, 10.1073/pnas.1600483113. 27325756 PMC4941449

[28] R. J. Dooling and S. D. Brown , “Speech Perception by Budgerigars (*Melopsittacus undulatus*): Spoken Vowels,” Perception & Psychophysics 47 (1990): 568–574, 10.3758/BF03203109. 2367177

[29] M. Hoeschele and W. T. Fitch , “Phonological Perception by Birds: Budgerigars Can Perceive Lexical Stress,” Animal Cognition 19, no. 3 (2016): 643–654, 10.1007/s10071-016-0968-3. 26914456 PMC4824828

[30] A. Hasegawa , K. Okanoya , T. Hasegawa , and Y. Seki , “Rhythmic Synchronization Tapping to an Audio‐Visual Metronome in Budgerigars,” Scientific Reports 1 (2011): 120, 10.1038/srep00120. 22355637 PMC3216601

[31] Y. Seki and K. Tomyta , “Effects of Metronomic Sounds on a Self‐Paced Tapping Task in Budgerigars and Humans,” Current Zoology 65, no. 1 (2019): 121–128, 10.1093/cz/zoy075. 30697247 PMC6347061

[32] M. Hoeschele and D. L. Bowling , “Sex Differences in Rhythmic Preferences in the Budgerigar (*Melopsittacus undulatus*): A Comparative Study With Humans,” Frontiers in Psychology 7 (2016): 1543, 10.3389/fpsyg.2016.01543. 27757099 PMC5047910

[33] F. Haiduk , D. L. Bowling , S. Afroozeh , J. Kopač , M. Azarian , and M. Hoeschele , “Female Budgerigars Attend to Musical Beat and Repetition, Humans Prioritise Their Combination,” Manuscript in revision.

[34] N. Wewhare and A. Krishnan , “Individual‐Specific Associations Between Warble Song Notes and Body Movements in Budgerigar Courtship Displays,” Biology Open 13, no. 10 (2024): bio060497, 10.1242/bio.060497. PMC11552614 39171537

[35] B. F. Brockway , “Roles of Budgerigar Vocalization in the Integration of Breeding Behavior,” in Bird Vocalizations: Their Relations to Current Problems in Biology and Psychology, ed. R. A. Hinda (Cambridge University Press, 1969), 131–158, 10.2307/4083992.

[36] H. Eda‐Fujiwara and H. Okumura , “The Temporal Pattern of Vocalizations in the Budgerigar (*Melopsittacus undulatus*),” Journal of the Yamashina Institute of Ornithology 24, no. 1 (1992): 18–31, 10.3312/jyio1952.24.18.

[37] N. Jacoby and J. H. McDermott , “Integer Ratio Priors on Musical Rhythm Revealed Cross‐Culturally by Iterated Reproduction,” Current Biology 27, no. 3 (2017): 359–370, 10.1016/j.cub.2016.12.031. 28065607

[38] T. C. Roeske , O. Tchernichovski , D. Poeppel , and N. Jacoby , “Categorical Rhythms Are Shared Between Songbirds and Humans,” Current Biology 30, no. 18 (2020): 3544–3555 e3546, 10.1016/j.cub.2020.06.072. 32707062 PMC7511425

[39] B. J. R. van der Vleuten , V. A. Hovenkamp , J. M. Varkevisser , and M. J. Spierings , “Context‐Dependent Rhythmicity in Chimpanzee Displays,” Proceedings of the Royal Society B Biological Sciences 291, no. 2036 (2024): 20242200, 10.1098/rspb.2024.2200. PMC11614530 39626754

[40] V. Eleuteri , J. van der Werff , W. Wilhelm , et al., “Chimpanzee Drumming Shows Rhythmicity and Subspecies Variation,” Current Biology 35, no. 10 (2025): 2448–2456 e2444, 10.1016/j.cub.2025.04.019. 40347944

[41] A. R. Lameira , M. E. Hardus , A. Ravignani , T. Raimondi , and M. Gamba , “Recursive Self‐Embedded Vocal Motifs in Wild Orangutans,” eLife 12 (2024): RP88348, 10.7554/eLife.88348. PMC10945596 38252123

[42] C. De Gregorio , T. Raimondi , V. Bevilacqua , et al., “Isochronous Singing in 3 Crested Gibbon Species (*Nomascus* spp.),” Current Zoology 70, no. 3 (2024): 291–297, 10.1093/cz/zoad029. 39035758 PMC11255994

[43] C. De Gregorio , M. Maiolini , T. Raimondi , et al., “Isochrony as Ancestral Condition to Call and Song in a Primate,” Annals of the New York Academy of Sciences 1537, no. 1 (2024): 41–50, 10.1111/nyas.15151. 38925552

[44] C. De Gregorio , D. Valente , T. Raimondi , et al., “Categorical Rhythms in a Singing Primate,” Current Biology 31, no. 20 (2021): R1379–R1380, 10.1016/j.cub.2021.09.032. 34699799

[45] M. Anichini , K. de Reus , T. A. Hersh , et al., “Measuring Rhythms of Vocal Interactions: A Proof of Principle in Harbour Seal Pups,” Philosophical Transactions of the Royal Society B: Biological Sciences 378, no. 1875 (2023): 20210477, 10.1098/rstb.2021.0477. PMC9985970 36871583

[46] V. Demartsev , M. Haddas‐Sasson , A. Ilany , L. Koren , and E. Geffen , “Male Rock Hyraxes That Maintain an Isochronous Song Rhythm Achieve Higher Reproductive Success,” Journal of Animal Ecology 92, no. 8 (2023): 1520–1531, 10.1111/1365-2656.13801. 36097377

[47] Y. Jadoul , T. Tufarelli , C. Coissac , M. Gamba , and A. Ravignani , “Hidden Assumptions of Integer Ratio Analyses in Bioacoustics and Music,” Annals of the New York Academy of Sciences (2025): 1‐16, 10.1111/nyas.70037. PMC12645274 41043082

[48] B. Sharma , X. Gao , K. Vijayan , X. Tian , and H. Li , “NHSS: A Speech and Singing Parallel Database,” Speech Communication 133 (2021): 9–22, 10.1016/j.specom.2021.07.002.

[49] D. C. Mann , W. T. Fitch , H. W. Tu , and M. Hoeschele , “Universal Principles Underlying Segmental Structures in Parrot Song and Human Speech,” Scientific Reports 11, no. 1 (2021): 776, 10.1038/s41598-020-80340-y. 33436874 PMC7804275

[50] H. W. Tu , E. W. Smith , and R. J. Dooling , “Acoustic and Perceptual Categories of Vocal Elements in the Warble Song of Budgerigars (*Melopsittacus undulatus*),” Journal of Comparative Psychology 125, no. 4 (2011): 420–430, 10.1037/a0024396. 22142040 PMC4497543

[51] T. Sainburg and A. Zorea , “Noisereduce: Domain General Noise Reduction for Time Series Signals,” Arxiv (2024),. 10.1038/s41598-025-13108-x PMC12373731 40847024

[52] T. Stivers , N. J. Enfield , P. Brown , et al., “Universals and Cultural Variation in Turn‐Taking in Conversation,” Proceedings of the National Academy of Sciences 106, no. 26 (2009): 10587–10592, 10.1073/pnas.0903616106. PMC2705608 19553212

[53] E. W. Large , I. Roman , J. C. Kim , et al., “Dynamic Models for Musical Rhythm Perception and Coordination,” Frontiers in Computational Neuroscience 17 (2023): 1151895, 10.3389/fncom.2023.1151895. 37265781 PMC10229831

[54] F. Pesarin and L. Salmaso , “The Permutation Testing Approach: A Review,” Statistica 70, no. 4 (2010): 481–509, 10.6092/issn.1973-2201/3599.

[55] S. M. Farabaugh and R. J. Dooling , “Acoustic Communication in Parrots: Laboratory and Field Studies of Budgerigars, *Melopsittacus undulatus* ,” in Ecology and Evolution of Acoustic Communication in Birds, ed. D. E. Kroodsma and E. H. Miller (Cornell University Press, 1996), 10.7591/9781501736957.

[56] J. T. Heaton , R. J. Dooling , and S. M. Farabaugh , “Effects of Deafening on the Calls and Warble Song of Adult Budgerigars (*Melopsittacus undulatus*),” Journal of the Acoustical Society of America 105, no. 3 (1999): 2010–2019, 10.1121/1.426734. 10089618

[57] H. Eda‐Fujiwara , A. Watanabe , and H. Okumura , “Effects of Deafening on the Temporal Pattern of Vocalizations in the Budgerigar (*Melopsittacus undulatus*),” Journal of Ethology 13, no. 2 (1995): 145–152, 10.1007/BF02350105.

[58] C. Tobin , A. Medina‐Garcia , G. M. Kohn , and T. F. Wright , “Does Audience Affect the Structure of Warble Song in Budgerigars (*Melopsittacus undulatus*)?,” Behavioural Processes 163 (2019): 81–90, 10.1016/j.beproc.2017.10.007. 29054444 PMC5906206

[59] A. G. Hile , N. T. Burley , C. B. Coopersmith , V. S. Foster , and G. F. Striedter , “Effects of Male Vocal Learning on Female Behavior in the Budgerigar, *Melopsittacus undulatus* ,” ethol 111, no. 10 (2005): 901–923, 10.1111/j.1439-0310.2005.01105.x.

[60] C. A. Marler and M. J. Ryan , “Origin and Maintenance of a Female Mating Preference,” Evolution; International Journal of Organic Evolution 51, no. 4 (1997): 1244–1248, 10.1111/j.1558-5646.1997.tb03971.x. 28565473

[61] P. O'Donald , “Sexual Selection by Female Choice in a Monogamous Bird: Darwin's Theory Corroborated,” Heredity 45 (1980): 201–2017, 10.1038/hdy.1980.61.

[62] R. A. Krebs and D. A. West , “Female Mate Preference and the Evolution of Female‐Limited Batesian Mimicry,” Evolution; International Journal of Organic Evolution 42 (1988): 1101–1104, 10.2307/2408927. 28581177

[63] S. M. Farabaugh , A. Linzenbold , and R. J. Dooling , “Vocal Plasticity in Budgerigars (*Melopsittacus undulatus*): Evidence for Social Factors in the Learning of Contact Calls,” Journal of Comparative Psychology 108, no. 1 (1994): 81–92, 10.1037/0735-7036.108.1.81. 8174348

[64] A. A. Nespor , M. J. Lukazewicz , R. J. Dooling , and G. F. Ball , “Testosterone Induction of Male‐Like Vocalizations in Female Budgerigars (*Melopsittacus undulatus*),” Hormones and Behavior 30, no. 2 (1996): 162–169, 10.1006/hbeh.1996.0020. 8797025

[65] S. D. Brown , R. J. Dooling , and K. E. O'Grady , “Perceptual Organization of Acoustic Stimuli by Budgerigars (*Melopsittacus undulatus*): III. Contact Calls,” Journal of Comparative Psychology 102, no. 3 (1988): 236–247, 10.1037/0735-7036.102.3.236. 3180731

[66] H. W. Tu , M. S. Osmanski , and R. J. Dooling , “Learned Vocalizations in Budgerigars (*Melopsittacus undulatus*): The Relationship Between Contact Calls and Warble Song,” Journal of the Acoustical Society of America 129, no. 4 (2011): 2289–2297, 10.1121/1.3557035. 21476684 PMC3087398

[67] F. Haiduk and W. T. Fitch , “Understanding Design Features of Music and Language: The Choric/Dialogic Distinction,” Frontiers in Psychology 13 (2022): 786899, 10.3389/fpsyg.2022.786899. 35529579 PMC9075586

[68] A. Ravignani and P. Norton , “Measuring Rhythmic Complexity: A Primer to Quantify and Compare Temporal Structure in Speech, Movement, and Animal Vocalizations,” Journal of Language Evolution 2, no. 1 (2017): 4–19, 10.1093/jole/lzx002.

[69] T. F. Wright and C. R. Dahlin , “Vocal Dialects in Parrots: Patterns and Processes of Cultural Evolution,” Emu—Austral Ornithology 118, no. 1 (2018): 50–66, 10.1080/01584197.2017.1379356. PMC6020700 29962561

