# Supplementary material for: Novel Approach to Inter‐Onset‐Interval Ratio Uncovers Music‐Like Rhythmic Patterns in Budgerigar (Melopsittacus undulatus) Warble Song
Source: Ann N Y Acad Sci. 2025 Dec 16;1557(1):e70164. doi: 10.1111/nyas.70164 (PMC7618608; doi:10.1111/nyas.70164)
Supplement: Supplementary file 1 — Supplementary Materials: nyas70164‐sup‐0001‐SuppMat.pdf [file NYAS-1557-0-s001.pdf]

Supplementary Materials

Novel approach to IOI-ratio uncovers structured rhythmic patterns similar to music in budgerigar (*Melopsittacus undulatus*) warble

Jeroen van der Aa[1*], Günther Koliander[2], W. Tecumseh Fitch[1] & Marisa Hoeschele[2*]

[1] Department of Behavioral and Cognitive Biology, University of Vienna, Vienna, Austria
[2] Acoustics Research Institute, Austrian Academy of Sciences, Vienna, Austria

* Correspondence:
Jeroen van der Aa and Marisa Hoeschele
jeroen.van.der.aa@univie.ac.at and marisa.hoeschele@oeaw.ac.at

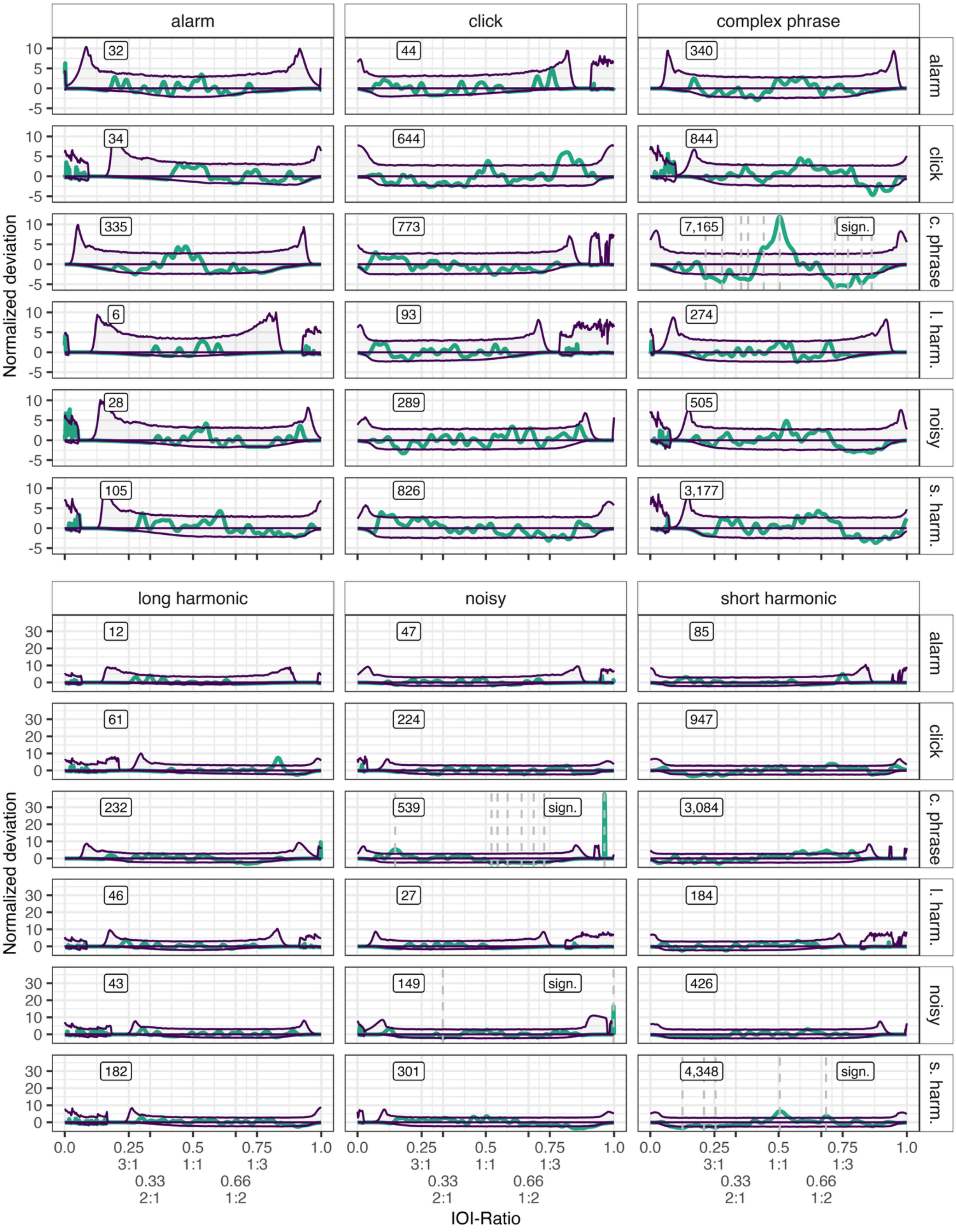

Figure S1. Normalized deviation distributions per warble element pair. The first element per pair is displayed per row, the second element per column. The thick (teal) line constitutes the observed data. The three (purple) thin lines constitute the permutation distributions: permutation average (middle line) ± confidence intervals (outer lines). Dashed lines demarcate local peaks that exceed the confidence interval.
Text boxes display underlying IOI-ratio sample size on the left, and significance status on the right per individual. Integer ratios are denoted below their respective IOI-ratio.

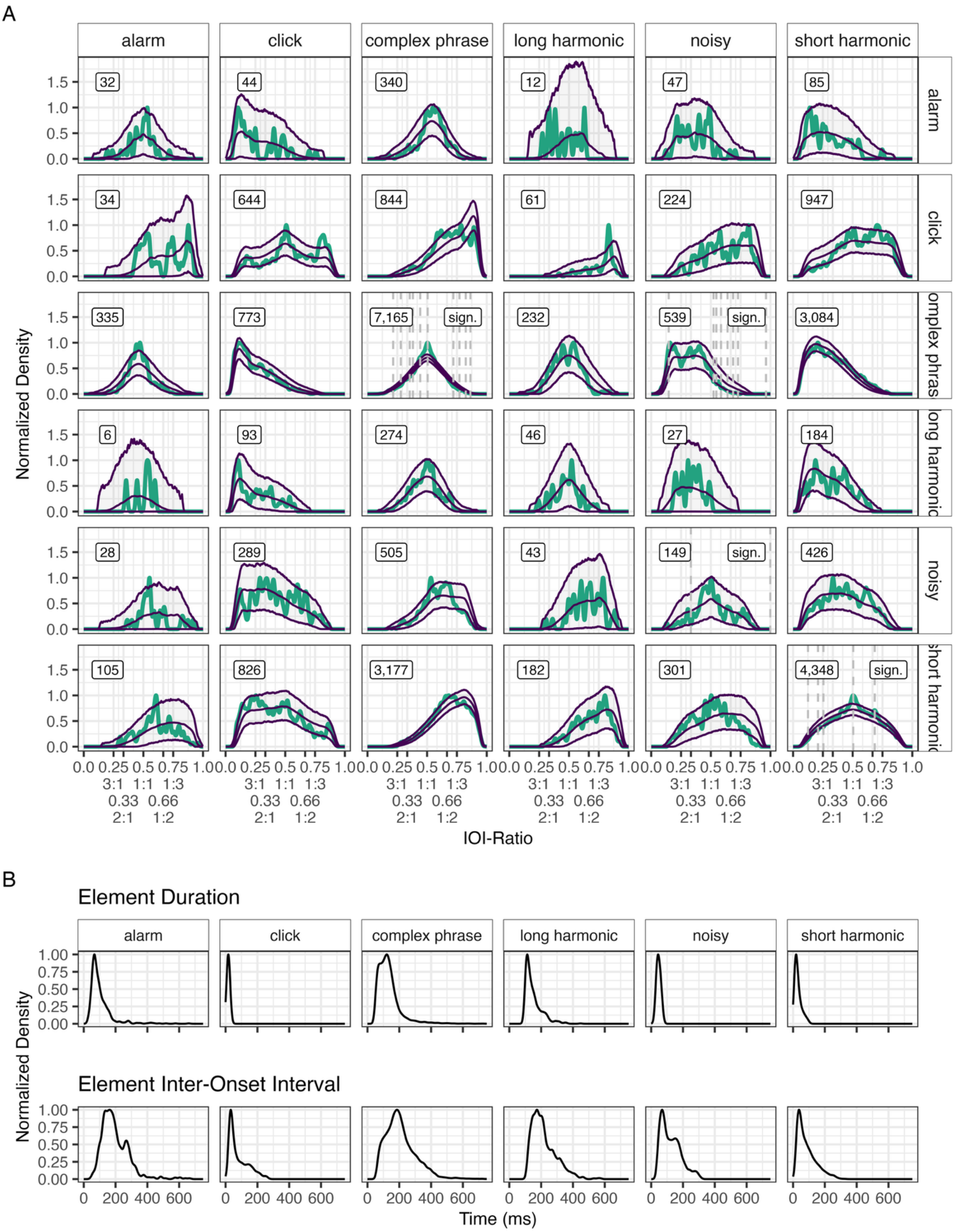

Figure S2. A) Full normalized density distributions per warble element pair. The first element per pair is displayed per row, the second element per column. The thick (teal) line constitutes the observed data. The three (purple) thin lines constitute the permutation distributions: permutation average (middle line) ± confidence intervals (outer lines). Dashed lines demarcate local peaks that exceed the confidence interval.
Text boxes display underlying IOI-ratio sample size on the left, and significance status on the right per individual. Integer ratios are denoted below their respective IOI-ratio.
B) Normalized densities of the element duration (top row) and element inter-onset interval (bottom row) per element.

Element 2	Element 1	Test Statistic	Max. Deviation
alarm	alarm	10.452977	6.278850
alarm	click	10.763594	3.502194
alarm	complex phrase	10.293794	4.489973
alarm	long harmonic	13.865248	4.173197
alarm	noisy	11.265105	7.715661
alarm	short harmonic	11.769990	4.285635
click	alarm	17.514246	4.787313
click	click	7.801844	6.040218
click	complex phrase	22.983172	3.000915
click	long harmonic	20.298990	2.249439
click	noisy	17.640020	3.758904
click	short harmonic	6.592025	3.998667
complex phrase	alarm	9.932486	2.998014
complex phrase	click	11.406796	5.645103
complex phrase	complex phrase	8.412234	11.890350 *
complex phrase	long harmonic	9.202656	2.710464
complex phrase	noisy	11.364158	4.840877
complex phrase	short harmonic	14.252546	4.339342
long harmonic	alarm	12.127370	4.140363
long harmonic	click	16.586453	7.401565
long harmonic	complex phrase	10.367448	9.493844
long harmonic	long harmonic	12.737873	3.480666
long harmonic	noisy	9.966837	3.536512
long harmonic	short harmonic	11.868346	2.782421
noisy	alarm	16.431827	3.800278
noisy	click	13.903398	3.301258
noisy	complex phrase	24.070569	37.676337 *
noisy	long harmonic	16.646443	1.888654
noisy	noisy	16.117648	16.492138 *
noisy	short harmonic	14.355573	3.547325
short harmonic	alarm	23.585313	4.948036
short harmonic	click	6.885304	3.799955
short harmonic	complex phrase	23.440762	4.612673
short harmonic	long harmonic	24.637652	3.164784
short harmonic	noisy	15.249040	2.985159
short harmonic	short harmonic	5.938487	6.455336 *

Table S1. Results of the statistical analyses per budgerigar element pair. The test statistic is equal to 99% of the maxima observed for the permutations. Maximum deviations of the observed data that are larger than the test statistic are demarcated with an asterisk (*).

ID		AFI	BEN	BOB	D18	ELV	GAN	HED	MAY	MER	PUC	PUF	RIC	TOP	UM1	AVG
Count	alarm	53	5	20	27	111	6	51	55	44	358	32	21	21	20	
	click	137	17	269	76	679	59	100	448	175	1,021	329	195	160	220	
	C. phrase	461	69	1,725	105	1,664	160	708	1,316	704	5,855	1,649	1,171	950	632	
	L. harm.	26	3	44	19	110	16	23	24	45	342	67	35	8	30	
	noisy	80	7	185	64	311	38	68	313	87	532	96	56	85	142	
	S. harm.	263	50	921	83	991	132	324	950	369	2,83	2,324	1,835	353	412	
	unkn.	105	23	411	109	400	48	150	364	172	1,153	455	505	137	85	
	total	1,125	174	3,575	483	4,266	459	1,424	3,470	1,596	12,091	4,952	3,818	1,174	1,541	
Percentage	alarm	4.71%	2.87%	0.56%	5.59%	2.60%	1.31%	3.58%	1.59%	2.76%	2.96%	0.65%	0.55%	1.23%	1.30%	2.30%
	click	12.18%	9.77%	7.52%	15.73%	15.92%	12.85%	7.02%	12.91%	10.96%	8.44%	6.64%	5.10%	9.33%	14.28%	10.60%
	C. phrase	40.98%	39.66%	48.25%	21.74%	39.01%	34.86%	49.72%	37.93%	44.11%	48.42%	33.30%	30.67%	55.43%	41.01%	40.40%
	L. harm.	2.31%	1.72%	1.23%	3.93%	2.58%	3.49%	1.62%	0.69%	2.82%	2.83%	1.35%	0.92%	0.47%	1.95%	1.99%
	noisy	7.10%	4.02%	5.17%	13.25%	7.29%	8.28%	4.78%	9.02%	5.45%	4.40%	1.94%	1.47%	4.96%	9.21%	6.17%
	S. harm.	23.38%	28.74%	25.76%	17.18%	23.23%	28.76%	22.75%	27.38%	23.12%	23.41%	46.93%	48.06%	20.60%	26.74%	27.60%
	unkn.	9.33%	13.21%	11.49%	22.57%	9.38%	10.46%	10.53%	10.49%	10.78%	9.54%	9.19%	13.23%	7.99%	5.52%	11.00%

Table S2. Distribution of elements produced for the 3 significant budgerigar individuals, both in absolute numbers (counts) and percentages. The average distribution is across all budgerigars recorded.

ID	Full data (no omission)		S-S omitted		C-C omitted	
	**Test Statistic**	**Max. Deviation**	**Test Statistic**	**Max. Deviation**	**Test Statistic**	**Max. Deviation**
ELV	5.714861	6.146125 *	5.707244	5.264774	5.838334	4.101945
MAY	5.604473	6.127583 *	5.289520	6.703747 *	5.436862	6.059194 *
PUC	5.141417	7.887247 *	5.143770	7.885933 *	5.152562	5.280907 *

Table S3. Results of the statistical analyses of the post-hoc analyses for the 3 significant budgerigar individuals, modelling the omission of the two element pairs, including the original full data. The test statistic is equal to 99% of the maxima observed for the permutations. Maximum deviations of the observed data that are larger than the test statistic are demarcated with an asterisk (*).
